# Assessment of photoplethysmography-based blood pressure determinations during long-term and short-term remote cardiac monitoring: the RECAMO study

**DOI:** 10.1093/ehjdh/ztaf027

**Published:** 2025-03-27

**Authors:** Mariska van Vliet, Jan J J Aalberts, Cora Hamelinck, Arnaud D Hauer, Dieke Hoftijzer, Stefan H J Monnink, Jurjan C Schipper, Jan C Constandse, Nicholas S Peters, Gregory Y H Lip, Steven R Steinhubl, Eelko Ronner

**Affiliations:** Department of Cardiology, Reinier the Graaf Hospital, Reinier de Graafweg 5, 2625 AD Delft, The Netherlands; Department of Cardiology, Reinier the Graaf Hospital, Reinier de Graafweg 5, 2625 AD Delft, The Netherlands; Department of Cardiology, Reinier the Graaf Hospital, Reinier de Graafweg 5, 2625 AD Delft, The Netherlands; Department of Cardiology, Reinier the Graaf Hospital, Reinier de Graafweg 5, 2625 AD Delft, The Netherlands; Department of Cardiology, Reinier the Graaf Hospital, Reinier de Graafweg 5, 2625 AD Delft, The Netherlands; Department of Cardiology, Reinier the Graaf Hospital, Reinier de Graafweg 5, 2625 AD Delft, The Netherlands; Department of Cardiology, Reinier the Graaf Hospital, Reinier de Graafweg 5, 2625 AD Delft, The Netherlands; Department of Cardiology, Reinier the Graaf Hospital, Reinier de Graafweg 5, 2625 AD Delft, The Netherlands; National Heart and Lung Institute, Imperial College London, London, UK; Department of Cardiac Electrophysiology, Imperial College Healthcare NHS Trust, London, UK; Liverpool Centre for Cardiovascular Science at the University of Liverpool, Liverpool John Moores University, Liverpool Heart and Chest Hospital, Liverpool, UK; Danish Centre for Health Services Research, Department of Clinical Medicine, Aalborg University, Aalborg, Denmark; Weldon School of Biomedical Engineering, Purdue University, West Lafayette, IN, USA; Department of Cardiology, Reinier the Graaf Hospital, Reinier de Graafweg 5, 2625 AD Delft, The Netherlands

**Keywords:** Blood pressure, Long-term evaluation, Ambulatory blood pressure monitoring, Photoplethysmography, Remote monitoring, Wearable diagnostics

## Abstract

**Aims:**

Cardiovascular diseases are a global health crisis, with hypertension as a significant risk factor. Traditional cuff-based blood pressure measurements have various limitations, prompting the exploration of photoplethysmography as an alternative for continuous monitoring. This study aimed to assess a cuff-calibrated wrist-worn photoplethysmography-based blood pressure device against European Society of Hypertension recommendations.

**Methods and results:**

The study assessed photoplethysmography-based blood pressure measurement stability over 28 days in 150 patients by comparing measurements of the wrist-worn photoplethysmography-based device against three daily automated reference blood pressure measurements. Additionally, awake–asleep blood pressure changes were analysed in 40 patients receiving 24-h ambulatory blood pressure monitoring. Data analysis included overall accuracy and recalibration needs during long-term monitoring, the accuracy of monitoring awake–asleep blood pressure changes, and resilience against hydrostatic pressure changes due to variations in device position. Across 28 days, mean errors of 3.84 mmHg (SD 4.46) for systolic and 4.08 mmHg (SD 3.97) for diastolic blood pressure were achieved. Before recalibration on Day 28, mean errors were 2.49 (SD 3.10) for systolic and 2.98 (SD 3.48) for diastolic blood pressure. Awake–asleep blood pressure change accuracy was demonstrated with mean errors of 2.36 (SD ± 2.40) for systolic and 2.17 (SD ± 2.13) for diastolic blood pressure. Hydrostatic pressure testing indicated resilience against changes in device position.

**Conclusion:**

The studied wrist-worn photoplethysmography-based device demonstrated accurate and stable blood pressure monitoring over 28 days, during awake–asleep blood pressure changes and hydrostatic pressure changes. These findings support the device’s potential for remote patient monitoring.

**Study registration:**

ClinicalTrials.gov identifier: NCT05899959.

## Graphical Abstract

**Graphical Abstract:**
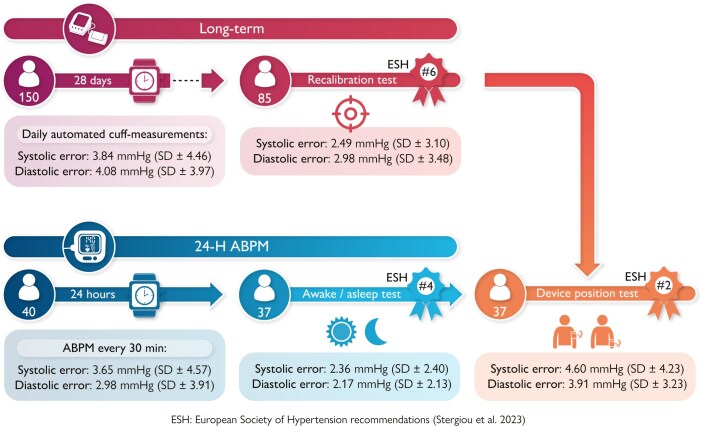

## Introduction

Cardiovascular diseases (CVDs) stand out as a global health crisis, being the primary cause of death worldwide.^1^ Among the known risk factors for CVD, high blood pressure (BP), also known as hypertension, holds the strongest causal association and is prevalent across a large portion of the population.^2^ In 2019, the leading risk factor globally for attributable deaths was high systolic BP (SBP).^3^ Research has shown that reducing high BP through various interventions can significantly decrease the risk of CVD.^2,4^ To achieve this, accurate monitoring and measurement of BP are essential and should be performed regularly.^4,5^

Traditional BP measurements are typically taken during occasional visits to outpatient clinics. This sporadic approach may not capture the actual variations in BP over time. Furthermore, these BP measurements can be altered by numerous factors, such as isolated office hypertension and masked hypertension.^6,7^ Various studies suggest that at-home BP measurements may be more accurate predictors of CVD than clinical BP measurements.^8,9^ Beyond just home BP spot checks, ambulatory BP monitoring (ABPM) has emerged as a more comprehensive alternative by providing insights into BP variations over a 24- to 48-h period, and including insights into nighttime BP variations, which are a significant predictor of overall cardiovascular, and non-cardiovascular mortality.^10^ Despite its utility, ABPM has drawbacks, such as a restricted time frame and recording frequency, high susceptibility to artefacts or technical issues, and manual data extraction requirements.^11^ Most importantly, poor tolerability due to device discomfort disrupting work and especially sleep significantly limits the frequency and duration of monitoring.^12,13^

Photoplethysmography (PPG) is emerging as a less burdensome alternative to traditional and ABPM-based remote BP monitoring. Leveraging light absorption and reflection principles, PPG offers a non-invasive and continuous method for tracking vital signs, typically through wearable devices.^14,15^ Furthermore, PPG-based BP monitoring can facilitate early intervention and personalized treatment strategies, ultimately improving patient outcomes and reducing healthcare burdens.^4,16^ Importantly, PPG avoids common sources of error in traditional methods, such as issues related to cuff inflation, limb compression, or incorrect cuff size selection.^17,18^

While numerous studies have shown the potential of PPG in measuring BP,^19–21^ the technology has substantial challenges, and despite dozens of developed devices, few have received regulatory approval and none have yet to enter into routine clinical practice. These shortcomings highlight the importance of validating novel PPG-based BP measurements with appropriate techniques,^22–24^ recognizing that traditional validation protocols only cover some of the necessary aspects of PPG-based BP monitoring.^22,25,26^ In 2014, the Institute of Electrical and Electronics Engineers (IEEE) introduced the first official standard for cuffless wearable BP devices,^27,28^ and in 2022, the first European standard for cuffless BP measurements was published.^29^ However, practical challenges have limited the widespread application of the IEEE standard, whereas the European standard focuses on validating continuous, rather than intermittent, monitoring devices.

Recognizing this gap, the European Society of Hypertension (ESH) recently issued recommendations for validating intermittent cuffless BP measuring devices, drawing on elements from the existing European Universal and IEEE standards.^30^ Despite these recent advancements, there has been limited research to evaluate PPG-based BP measurements against these new ESH recommendations. Therefore, the present study aimed to assess a cuff-calibrated intermittent PPG-based wearable BP monitoring device on long-term measurement stability, accuracy before recalibration, resistance to hydrostatic pressure changes, and accuracy of measuring awake–asleep BP changes.

## Methods

### Study design and participants

This prospective single-centre study involved adult patients undergoing 24-h Holter or ABPM monitoring at Reinier de Graaf Gasthuis, a Dutch teaching hospital, from June to December 2023. Individuals of 18 years or older, able to provide consent, and receiving either 24-h Holter or ABPM monitoring by doctor prescription were eligible for inclusion. Exclusions included individuals unable to wear a wristband, receive cuff BP measurements, or those pregnant, breastfeeding, with already diagnosed chronic atrial fibrillation (AF), with arm circumferences outside 22–42 cm, unable to consent, or inability to follow study procedures. All participants provided written informed consent. The study, a follow-up to the MULTI-VITAL trial (NCT05566886), registered as the RECAMO study (NCT05899959 and NL83281.000.22), was approved by a regional ethics committee and adhered to the principles of the Declaration of Helsinki.

**Figure 1:**
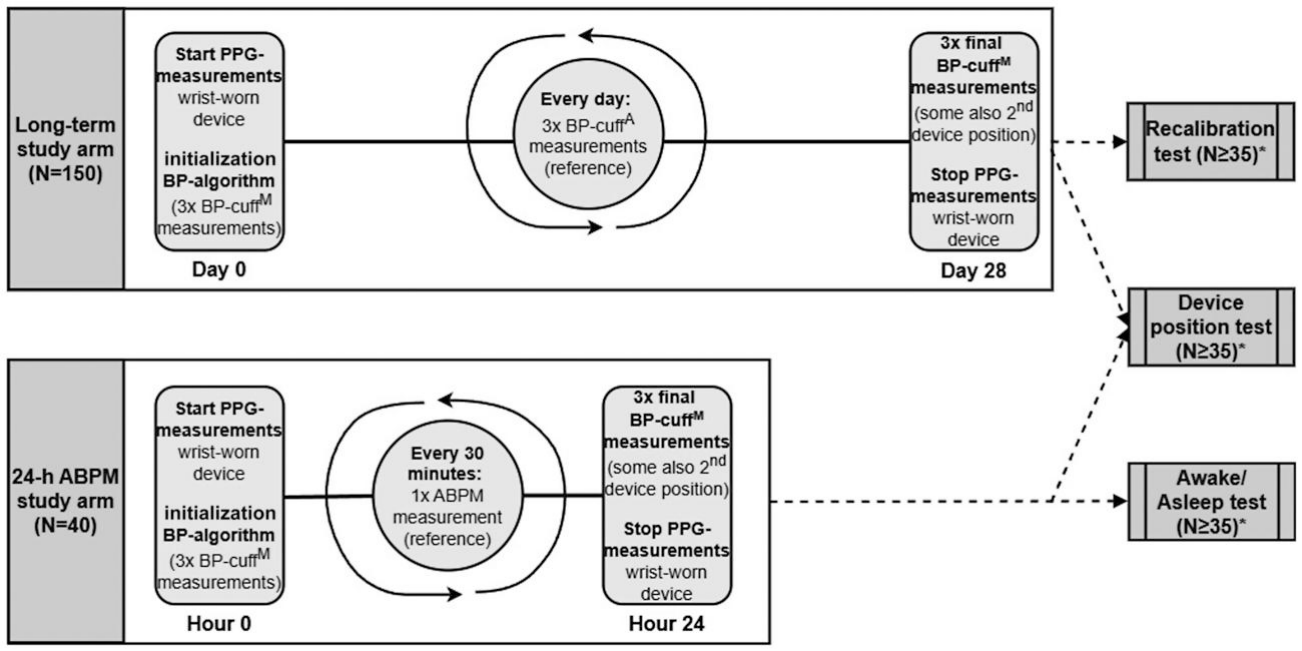
Overview of the study interventions and participant flow across both the long-term and short-term study arms, illustrating the timeline of the three accuracy tests based on the European Society of Hypertension recommendations for validating intermittent, cuff-calibrated blood pressure monitoring devices. The long-term study arm involves inclusion of 150 participants who received a wrist-worn photoplethysmography device and three manual cuff measurements on Day 0, followed by 28 days of daily automated cuff measurements at home. The study concludes with three final manual cuff measurements just before recalibration, as instructed by the manufacturer, on Day 28. This study arm provides data for the European Society of Hypertension recalibration test. The short-term study arm includes 40 participants who undergo 24-h ambulatory blood pressure monitoring with automated cuff measurements taken every 30 min. These participants also received a wrist-worn photoplethysmography device and three manual cuff measurements on Hour 0, followed by 24 h of intermittent blood pressure monitoring. Final measurements, including three manual cuff readings, were performed after 24 h. This group’s awake and asleep BP data were used to assess the European Society of Hypertension awake–asleep test. In both study arms, participants were also asked to complete a final blood pressure measurement with the arm of the investigation device in a ≥20 cm change in position for the European Society of Hypertension device position test. ABPM, ambulatory blood pressure monitoring; BP, blood pressure; PPG, photoplethysmography.

To evaluate the performance of the cuff-calibrated intermittent PPG-based wearable BP monitoring device in accordance with the latest ESH recommendations,^30^ two study arms (long-term and short-term) were implemented. These arms enabled the assessment of three key tests outlined by the ESH: the recalibration test, the device position test, and the awake–asleep test. A comprehensive overview of the study interventions and tests, arranged along a timeline, is shown in *Figure 1*. The endpoints for all tests were based on Criterion 1, as specified in the European Universal Standard,^25,26^ requiring a mean error of less than ±5.0 mmHg and a standard deviation (SD) below ±8.0 mmHg.

Specifically, the recalibration test assesses the device’s stability over time by evaluating its accuracy immediately before the next recalibration is due, as determined by the manufacturer (here: after 28 days). The device position test evaluates the device’s robustness against changes in hydrostatic pressure by testing its performance when positioned with at least a 20 cm vertical distance shift. Lastly, the awake–asleep test serves as the primary evaluation of the device’s ability to accurately detect BP changes between wakefulness and sleep, comparing results between the investigational device and a reference device. Throughout all tests, the subject, study design, and analysis criteria proposed by the ESH were adhered to as closely as possible.

### Study procedures and data collection

Participants were equipped with a wrist-worn PPG device (CardioWatch 287-2, Corsano Health B.V., the Hague, the Netherlands) on their non-dominant wrist directly following 24-h Holter or 24-h ABPM placement.^31^ This device is intended for intermittent BP monitoring by providing SBP and diastolic BP (DBP) values every 30 min. The PPG-based BP algorithm was initiated through three sequential cuff measurements on the opposite arm using a validated automated BP monitor (model TMB-2084-A, Zhongshan Transtek Electronics Co., Ltd).^32^ Patients who received a 24-h Holter were instructed to wear the wrist-worn PPG device continuously for 28 days (long-term study arm). Patients receiving a 24-h ABPM only wore the wrist-worn PPG device throughout 24 h (24-h study arm). During these periods, PPG data were continuously collected at 128 Hz and transmitted to a secure cloud. In the long-term arm, participants also performed daily home cuff BP measurements, with the average serving as a reference standard; the 24-h ABPM group did not perform additional home measurements.

Before and after the measurement periods, two trained observers conducted three sets of sequential BP measurements using a mercury sphygmomanometer, assessing SBP and DBP using the Phase One and Phase Five Korotkoff sounds, respectively. Measurements were repeated if they differed by more than 4 mmHg between observers.

At the start of the study period, baseline parameters, including skin type (Fitzpatrick scale) and arm hair density, were collected for all patients. The researchers documented all adverse events and study withdrawals. In case of study withdrawal or insufficient data quality, participants were replaced following the study protocol.

### Device position subgroup

A subgroup of the RECAMO study underwent additional two-observer BP measurements with the arm of the wrist-worn PPG device hanging freely. This test was used to evaluate the device’s response to hydrostatic pressure differences, as the ESH recommended in Stergiou *et al*.^30^

### Data processing and statistical analysis

The European Universal Standard and ESH suggest minimum sample sizes of 85 and 35 patients, respectively.^25,30^ However, as another primary outcome of the study was the evaluation of AF detection and quantification across 28 days, we required a sample size of 125 patients for the long-term study arm based on the AF outcome measures (see Supplementary material online, *File S1*). Factoring in an expected attrition rate of 20%, 150 patients were included in this study arm. For the 24-h study arm, using the ESH recommendation and a 15% attrition rate, we determined a sample size of 40 patients.^30^

The investigation devices use a PPG sensor to measure BP non-invasively. It emits light through LEDs and detects the reflected light with photodiodes, which varies based on blood flow in the arteries. The resulting PPG signal contains pulsations linked to blood volume changes during each heartbeat. The bracelet determines BP by analysing this waveform and comparing it to data from previous patients using an artificial intelligence (AI)-based algorithm.

As described earlier, features are extracted from the PPG segments, which are used in an AI-based model together with cuff-based initialization measurements as well as demographic information.^33^ To get a valid prediction, statistical features, time and frequency domain features, demographic features, first/second derivative features, width-related PPG features, and features from the PPG signal are used.

The AI model, a support vector machine with a radial basis function kernel, learns from data that is structured with the features mentioned above and discovers patterns in this data. The trained model gives weights to each feature and, in this way, ranks the feature from most important to least important. Using the extracted features and the initialization measurements, the model then outputs a SBP and DBP value for that segment.

### Long term

Long-term performance was assessed against the European Universal Standard criteria, evaluating mean difference and SD, for SBP and DBP individually.^25,26^ To do so, the average of the three reference SBP and DBP readings were compared with the average CardioWatch 287-2 (CW2) SBP and DBP across a 2-min measurement (sample frequency 128 Hz) at the corresponding time.

The correlation and Bland–Altman figures were determined according to Bland *et al*.^34^ All calculations were performed for all paired data points, for the two-observer simultaneous measurements at the study’s end (recalibration test), and after a change in device position (device position test).

Additionally, a sub-analysis calculated mean error and SD across four BP categories (normal, prehypertension, and Stage 1 and Stage 2 hypertension) as per IEEE standards.^27,28^

### Twenty-four-hour ambulatory blood pressure monitoring

For the 24-h ABPM, we analysed absolute BP differences and awake–asleep BP change differences (mean ± SD) throughout the 24-h cycle, and during awake and asleep states, across the general population and per nighttime dipper category: reverse dipper/rise (≤0% dip); non-dipper (≤10% dip), dipper (10 ≤ 20% dip), and extreme dipper (>20% dip). The SBP and DBP are determined by the 24-h ABPM with a 30-min measurement interval. The 24-h reference determined the BP with a 30-min measurement interval. To compare the test and reference device, awake–asleep BP changes were evaluated in light of the European Universal Standard.^28,30,33^ Furthermore, Bland–Altman figures and correlation plots were determined according to Bland *et al*.^34^ for the awake–asleep BP changes and the BP measurements across the entire 24-h period.

## Results

Consent was obtained from a total of 215 patients, but 10 patients withdrew from the study due to either too high a study burden (*n* = 5), allergic reaction to the bracelet strap (*n* = 4), or hospitalization (*n* = 1). All allergic reactions were caused by known allergies and caused mild irritation and redness at the location of the bracelet strap (material: PET-Polyester) only. For 13 patients in the long-term study arm, the majority of PPG data was of poor quality or was lost due to Wi-Fi connection issues. Furthermore, reference ABPM data were unavailable for two patients in the 24-h study arm. These 25 patients were replaced according to the study protocol.

Of the 215 consented participants, seven experienced adverse events, including an allergic reaction to the bracelet strap (*n* = 5), concussion (*n* = 1), and hospitalization due to COVID-19 infection (*n* = 1). For the long-term study arm, 150 participants contributed 4037 paired reference and CW2 BP measurements across the entire study period. For the 24-h study arm, 40 participants provided 987 paired BP measurements. *Table 1* provides patient characteristics for the long-term and 24-h study arms separately. Subgroup characteristics are described in Supplementary material online, *Tables S1* and *S2* (see Supplementary material online, *File S2*).

**Table 1:** Patient characteristics were individually reported for the long-term and 24-h study arms

	Long-term (*n* = 150)	24-h ABPM (*n* = 40)
Age, years	64 ± 12	68 ± 10
Females, *n* (%)	61 (41%)	16 (40%)
Weight, kg	84 ± 18	85 ± 20
Height, cm	176 ± 12	173 ± 10
Body mass index, kg/m^2^	28 ± 10	29 ± 7
Skin colour (Fitzpatrick), *n* (%)^39^		
Class I and II	138 (92%)	36 (90%)
Class III and IV	12 (8%)	4 (10%)
Class V and VI	0 (0%)	0 (0%)
Arm hair density, *n* (%)^40^		
Class nill/sparse	99 (66%)	32 (80%)
Class moderate	45 (30%)	6 (15%)
Class high	4 (4%)	2 (5%)
Clinic systolic blood pressure, mmHg	129 ± 80	139 ± 19
Clinic diastolic blood pressure, mmHg	80 ± 13	83 ± 11
Clinical hypertension, *n* (%)	47 (31%)	18 (45%)
(average SBP ≥ 140/DBP ≥ 90 mmHg)		
Use of ≥1 blood pressure lowering agent, *n* (%)	15 (10%)	NA
24-h ABPM hypertension, *n* (%)	NA	17 (43%)
(average SBP ≥ 130/DBP ≥ 80 mmHg)		

ABPM, ambulatory blood pressure monitoring; DBP, diastolic blood pressure; *n*, number of patients; NA, not applicable; SBP, systolic blood pressure; SD, standard deviation.

Subject characteristics adhered to the recommendations set by the ESH for all three tests performed, including a sample size > 35 participants, ages between 18 and 80 years, at least 30% males and 30% females, and reference BP distributions in accordance with the Universal Standard.^24,25^ An exception was made for the number of participants in the lowest BP range (5% with SBP ≤ 100 mmHg and 5% with DBP ≤ 60 mmHg) due to 31% of the patients being hypertensive. However, when considering revised limits of SBP ≤ 110 mmHg and DBP ≤ 70 mmHg, the 5% criteria were met. Additionally, for the short-term study arm, all specific ESH criteria regarding average 24-h ambulatory cuff SBP and DBP were met, including ≥30% of participants with average reference ambulatory SBP ≥ 130 mmHg and <120 mmHg, and DBP ≥ 80 mmHg and <70 mmHg across 24 h.^30^

### Long-term study arm

**Figure 2:**
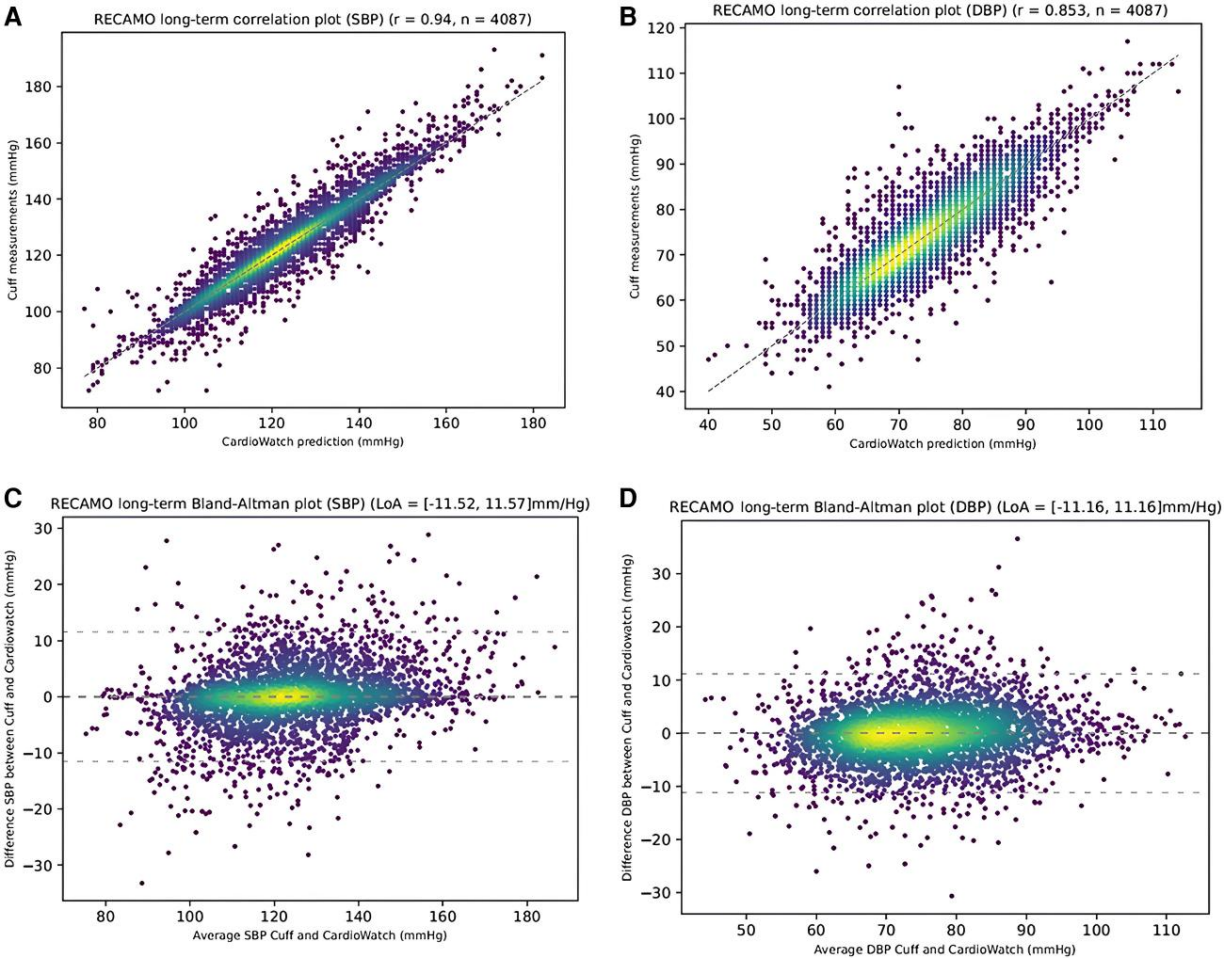
Visualization of the correlation between the investigational and reference (*A*) systolic and (*B*) diastolic blood pressure as well as Bland–Altman analysis for (*C*) systolic and (*D*) diastolic blood pressure investigational and reference comparisons using density plots (high to low density = light to dark colour). DBP, diastolic blood pressure; SBP, systolic blood pressure.

Across the entire 28-day period, the mean error and corresponding SD for the PPG-based compared with the cuff-based BP determinations were 3.84 (SD 4.46) for SBP and 4.08 (SD 3.97) for DBP. Bland–Altman and correlation plots for the 28-day measurement period are provided in *Figure 2*.

Measurement accuracy throughout the 28-day period was similar across all BP subcategories:

- Normal BP (*n* = 1635): systolic 3.88 (SD 4.67) and diastolic 3.41 (SD 3.33);
- Prehypertension (*n* = 1997): systolic 3.77 (SD 4.09) and diastolic 4.60 (SD 4.18);
- Stage 1 hypertension (*n* = 410): systolic 4.00 (SD 5.12) and diastolic 4.21 (SD 4.59); and
- Stage 2 hypertension (*n* = 45): systolic 4.32 (SD 5.96) and diastolic 4.07 (SD 6.03).

**Figure 3:**
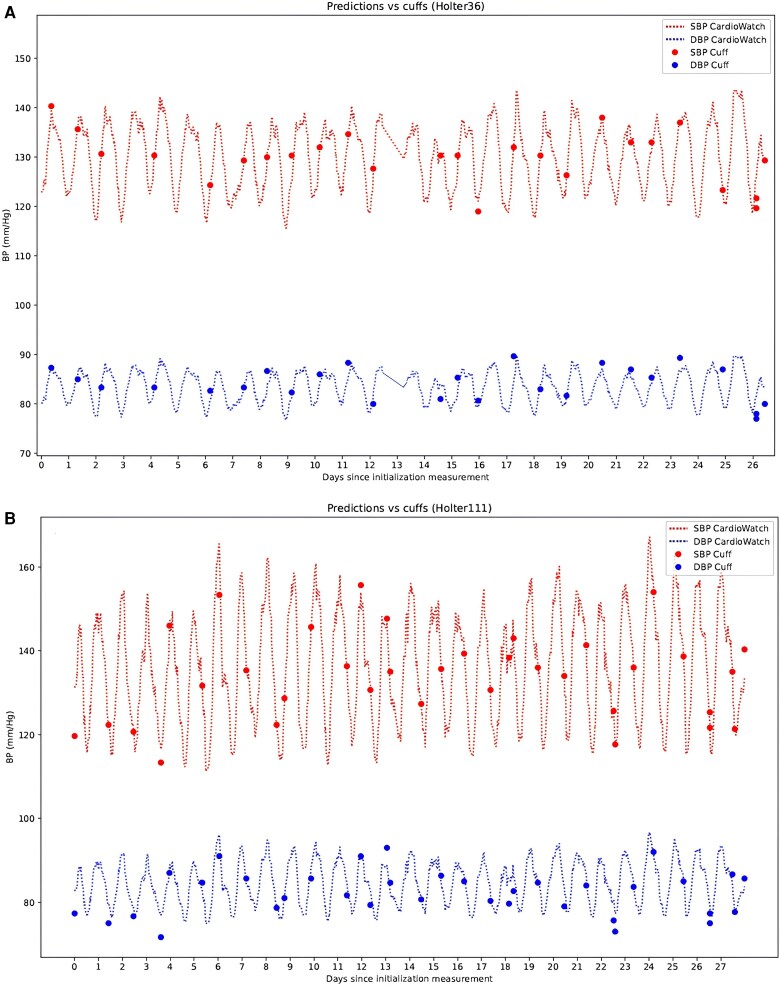
Photoplethysmography-based blood pressure predictions compared with cuff-based reference measurements for two patients (*A*) 36 and (*B*) 111, across the 28-day measurement period. BP, blood pressure; DBP, diastolic blood pressure; SBP, systolic blood pressure.

*Figure 3* additionally provides an overview of the PPG-based BP predictions and average cuff-based reference measurements across 28 days in two patients.

### Recalibration test

For a subgroup of 85 patients, the mean error immediately before the recommended recalibration on Day 28 was 2.49 (SD 3.10) for SBP and 2.98 (SD 3.48) for DBP. Corresponding Bland–Altman plots are provided in Supplementary material online, *Figure S1* (see Supplementary material online, *File S2*).

### Twenty-four-hour ambulatory blood pressure monitoring study arm

Across the 24 h, the average absolute BP difference between the investigational and reference device was 3.65 (SD ± 4.57) for SBP and 2.98 (SD ± 3.91) for DBP. Average absolute BP differences were similar for subsets of awake and asleep measurements (*awake* SBP: 3.59 ± 4.60 and DBP: 2.99 ± 3.93; *asleep* SBP: 3.78 ± 4.46 and DBP: 2.97 ± 3.84). Corresponding Bland–Altman and correlation plots are provided in Supplementary material online, *Figure S2* (see Supplementary material online, *File S2*).

The absolute difference in average awake–asleep BP change was 2.36 (SD ± 2.40) for SBP and 2.17 (SD ± 2.13) for DBP. Of the patients with nighttime ABPM data, 8.1% were extreme dippers, 40.5% dippers, 35.1% non-dippers, and 16.2% reverse dippers. Across the dipper categories, the absolute difference in average awake–asleep BP change ranged 1.17–3.27 mmHg (SD 1.09–2.90) for SBP and 0.78–2.85 mmHg (SD 0.10–2.41) for DBP (*Table 2*). Corresponding Bland–Altman and correlation plots are provided in Supplementary material online, *Figure S3* (see Supplementary material online, *File S2*).

**Table 2:** The absolute difference in average awake-to-asleep blood pressure change for the investigational compared with the reference device overall and per nighttime dipper category

Dipper category	Patients per dipper category	Systolic BP (mmHg)	Diastolic BP (mmHg)
Extreme dipper (>20%)	*n* = 3	2.71 (SD ± 1.65)	0.78 (SD ± 0.10)
Dipper (>10% and ≤20%)	*n* = 15	3.27 (SD ± 2.90)	2.83 (SD ± 2.10)
Non-dipper (>0% and ≤10%)	*n* = 13	1.17 (SD ± 1.09)	1.4 (SD ± 2.06)
Reverse dipper (≤0%)	*n* = 6	2.48 (SD ± 2.77)	2.85 (SD ± 2.41)
Overall (all %)	*n* = 37	2.36 (SD ± 2.40)	2.17 (SD ± 2.13)

BP, blood pressure; *n*, number of patients; SD, standard deviation.

### Device position subgroup

**Figure 4:**
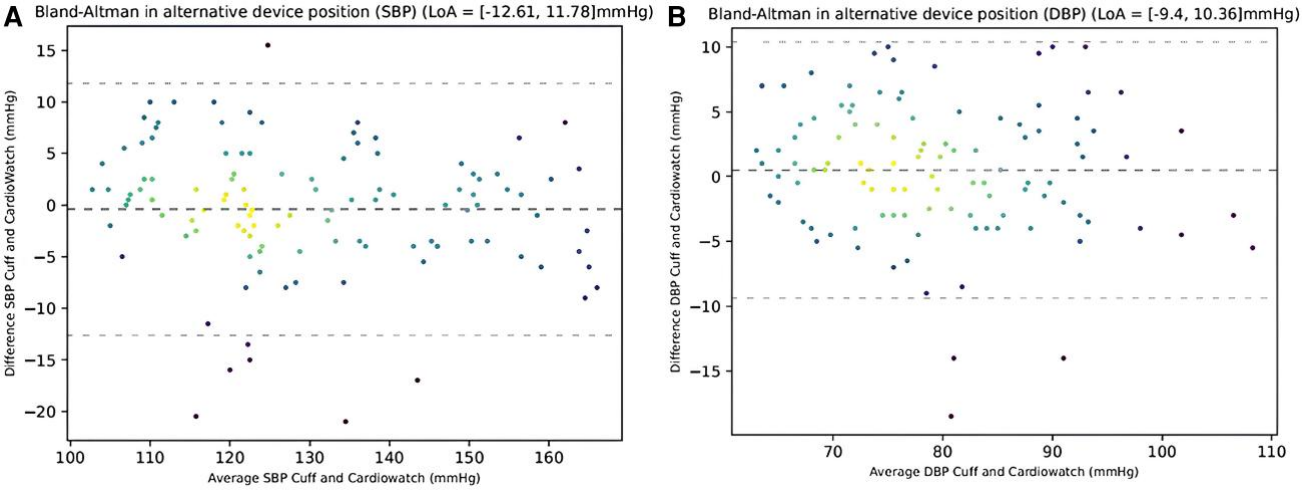
Visualization of Bland–Altman analysis for the investigational and reference (*A*) systolic and (*B*) diastolic blood pressure. DBP, diastolic blood pressure; SBP, systolic blood pressure.

For a subgroup of 37 patients, paired BP measurements were performed in a second device position to assess accuracy after a change in hydrostatic pressure. The mean error observed here was 4.60 (SD 4.23) for SBP and 3.91 (SD 3.23) for DBP. Corresponding Bland–Altman plots are provided in *Figure 4*.

## Discussion

The findings in this study demonstrate that the wrist-worn PPG device can provide accurate and stable BP monitoring over 28 days, spanning various BP ranges. The mean error and SD consistently fell below the threshold values outlined by Criteria 1 and 2 of the European Universal Standard, confirming that recalibration sooner than the 28-day timeframe is unnecessary. Second, measurements remained stable throughout nocturnal monitoring compared with a 24-h ABPM reference. Specifically, awake–asleep BP changes and various BP dipping patterns could be accurately monitored. Blood pressure monitoring showed lower errors for non-dippers compared with dippers, extreme dippers, and reverse dippers; however, differences between subgroups were small (≤2.1 mmHg) and remained below the outlined criteria for all subgroups, demonstrating sensitivity to nocturnal BP changes. Finally, the device was robust against variations in hydrostatic pressure. These results confirm the accuracy of the device in the recalibration test, device position test, and awake/asleep test, as recommended by the ESH, in accordance with the criteria outlined in the European Universal Standard.

Previous research has found PPG to be a promising tool for continuous short-term BP monitoring, typically immediately following calibration.^19–21^ However, limited research has been performed to investigate PPG-based BP algorithms’ long-term (≥28 days) stability. Overall, the results reported in this study align with those reported in the existing literature. Wang *et al*.^35^ demonstrated estimation errors of −3.38 ± 7.57 mmHg for SBP and −1.62 ± 4.99 mmHg for DBP after 5 weeks in 30 subjects. In two other studies, Yoon *et al*.^36^ and Vybornova *et al*.,^37^ lower mean errors together with higher SDs were demonstrated (systolic: 0.1 ± 8.8 and 0.46 ± 7.75 mmHg; diastolic: −2.4 ± 7.6 and 0.39 ± 6.86 mmHg, respectively); these studies were conducted across 4 weeks in 15 and 86 patients, respectively. Finally, study of Miao *et al*.^38^ was the only study to evaluate the performance of two PPG-based algorithms across 6 months, demonstrating low estimation errors (systolic: −1.27 ± 5.98 and −1.15 ± 5.79 mmHg; diastolic: −1.38 ± 5.49 and −1.19 ± 5.29 mmHg) for both a multivariate linear regression and a support vector regression model, respectively. Nonetheless, this research was conducted among healthy volunteers across only 10 subjects.

Recently, the sufficiency of the validation techniques employed in assessing PPG-based BP determinations has been debated. In response, the ESH has published updated recommendations.^23,30^ To the best of our knowledge, the present study represents the most extensive investigation of its kind, as it addressed four out of six elements recommended by the ESH. Notably, no exclusion criteria were imposed based on cardiac conditions other than already diagnosed chronic AF before inclusion, suggesting that the study may offer a more accurate representation of the CVD patient population than other studies that do exclude based on cardiac conditions. Nonetheless, this paper remains to be only one of the first to tackle the issues of conventional BP monitoring methods. Therefore, further evaluations will have to be performed to eventually allow the standardization of PPG-based BP measurements in clinical practice.

### Limitations

There are several limitations to this study. First, it omitted the exercise and treatment tests recommended by the ESH. However, an initial test during motion was addressed in the 24-h ABPM study arm. Additional studies will be performed to evaluate further the influence of exercise and treatment with BP-lowering medication on the accuracy of the wrist-worn PPG-based BP monitoring device.

Second, in this long-term study, only three consecutive cuff measurements were conducted each day, limiting the variation in measurement times. Therefore, tracking of BP variations throughout the day and night was further investigated using the 24-h ABPM study arm.

Third, the patient population under consideration constituted a subset of CVD patients, specifically those receiving a 24-h Holter. This may have influenced the study results as these patients are often subject to arrhythmias. Nevertheless, efforts were made to encompass patients across a spectrum of BP ranges and patient characteristics. Despite these efforts, the vast majority of participants had lighter skin colours, preventing us from ruling out the influence of skin pigmentation on the accuracy of PPG-based BPs. Additionally, as the device is intended for use in a hypertensive population, and given the limited data available for this subgroup, further research is necessary to evaluate its performance specifically in hypertensive patients. Future studies should aim for a more diverse and representative population, ensuring broader inclusion across skin pigmentation and various BP subgroups.

Despite these limitations, this study significantly contributes to our understanding of the accuracy and long-term stability of BP determinations through a wrist-worn PPG device, representing a pivotal step toward its application in remote patient monitoring.

In contrast to many cuffless PPG-based BP measurement devices that focus on assessing BP,^35–37^ the device evaluated in this study offers the capability to measure multiple other vital parameters. This multi-parameter functionality presents the potential for early detection of various health conditions and guides treatments efficiently and continuously. Consequently, devices such as the one under evaluation in this study may play a pivotal role in enabling early intervention and personalized treatment strategies, ultimately enhancing patient outcomes and alleviating healthcare burdens.^16^

Finally, while our findings support the reliability of PPG-based BP monitoring, future work should explore its performance during physical activity and BP medication changes. Furthermore, if PPG-derived BP monitoring is to be used in clinical practice, it will need to be further determined how the measurements derived from the continuous PPG trace relate to conventional sphygmomanometer-derived BPs and hence the clinical guidelines.

In conclusion, this study demonstrates the wrist-worn PPG device’s capacity for accurate and stable BP monitoring over 28 days, encompassing various BP ranges and body positions as well as awake–asleep BP changes.

## Supplementary Material

ztaf027_Supplementary_Data

## Data Availability

The analysed data will be made available by the corresponding author upon reasonable request.
