# Supplementary material for: Assessment of photoplethysmography-based blood pressure determinations during long-term and short-term remote cardiac monitoring: the RECAMO study
Source: Eur Heart J Digit Health. 2025 Mar 27;6(4):763–71. doi: 10.1093/ehjdh/ztaf027 (PMC12282382; doi:10.1093/ehjdh/ztaf027)
Supplement: ztaf027_Supplementary_Data [file ztaf027_supplementary_data.zip › Supplementary file 1.docx]

# Supplementary file 1

## Sample size calculation (atrial fibrillation)

Sample size calculation for the assessment of atrial fibrillation detection is based on a previous study that we conducted at an outpatient referral clinic, where we observed an atrial fibrillation incidence of 3% after 48 hours of EKG monitoring. To observe a 7% absolute increase in atrial fibrillation detection with a power of 0.80 at a two-sided α = 0.05, a total of 125 patients is required (McNemar test on dependent proportions). Assuming an attrition rate of 20%, a total of 150 patients will be included.
