# Supplementary material for: Assessment of photoplethysmography-based blood pressure determinations during long-term and short-term remote cardiac monitoring: the RECAMO study
Source: Eur Heart J Digit Health. 2025 Mar 27;6(4):763–71. doi: 10.1093/ehjdh/ztaf027 (PMC12282382; doi:10.1093/ehjdh/ztaf027)
Supplement: ztaf027_Supplementary_Data [file ztaf027_supplementary_data.zip › Supplementary file 2.docx]

# Supplementary file 2

| **Supplementary table 1.** Patient characteristics of patients included in the recalibration test (n=85) | |
| --- | --- |
| Age, years | 63 ± 12 |
| Females, N (%) | 41 (48%) |
| Weight, kg | 84 ± 19 |
| Height, cm | 174 ± 12 |
| Body mass index, kg/m^2^ | 28 ± 12 |
| Skin colour (Fitzpatrick), N (%) [33]  Class I-II  Class III-IV  Class V-VI | 80 (94%)  5 (6%)  0 (0%) |
| Arm hair density, N (%) [34]  Class Nill/Sparse  Class Moderate  Class High | 60 (71%)  21 (25%)  4 (5%) |
| Clinic systolic blood pressure, mmHg | 129 ± 19 |
| Clinic diastolic blood pressure, mmHg | 81 ± 12 |
| Clinical hypertension, N (%)  *(Average SBP ≥ 140 / DBP ≥ 90 mmHg)* | 27 (32%) |
| **Abbreviations** SD: standard deviation; N: number of patients; SBP: systolic blood pressure; DBP: diastolic blood pressure | |

| **Supplementary table 2.** Patient characteristics of patients included in the device position test (n=37) | |
| --- | --- |
| Age, years | 63 ± 13 |
| Females, N (%) | 17 (46%) |
| Weight, kg | 82 ± 17 |
| Height, cm | 175.5 ± 10 |
| Body mass index, kg/m^2^ | 27 ± 5 |
| Skin colour (Fitzpatrick), N (%) [33]  Class I-II  Class III-IV  Class V-VI | 35 (95%)  2 (5%)  0 (0%) |
| Arm hair density, N (%) [34]  Class Nill/Sparse  Class Moderate  Class High | 27 (73%)  9 (24%)  1 (3%) |
| Clinic systolic blood pressure, mmHg | 131 ± 83 |
| Clinic diastolic blood pressure, mmHg | 20 ± 11 |
| Clinical hypertension, N (%)  *(Average SBP ≥ 140 / DBP ≥ 90 mmHg)* | 11 (30%) |
| **Abbreviations** SD: standard deviation; N: number of patients; SBP: systolic blood pressure; DBP: diastolic blood pressure | |


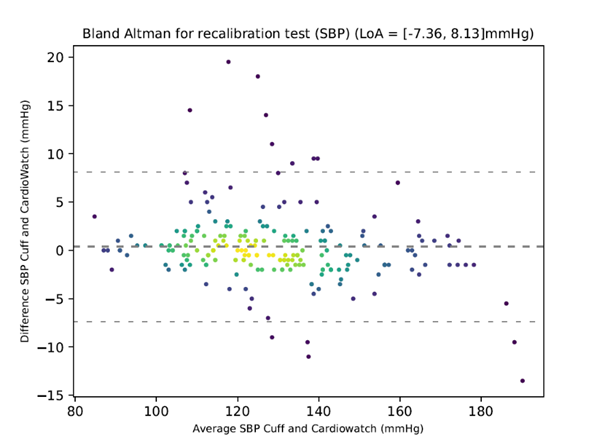

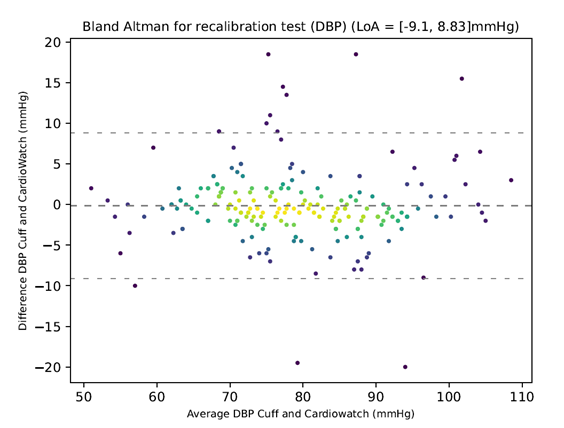


**Supplementary figure 1.** Visualisation of Bland-Altman analysis for the investigational and reference **a)** systolic and **b)** diastolic blood pressure determinations on day 28 of the study period (recalibration test) using density plots (high to low density = yellow – green – blue – purple).

**B**

**A**


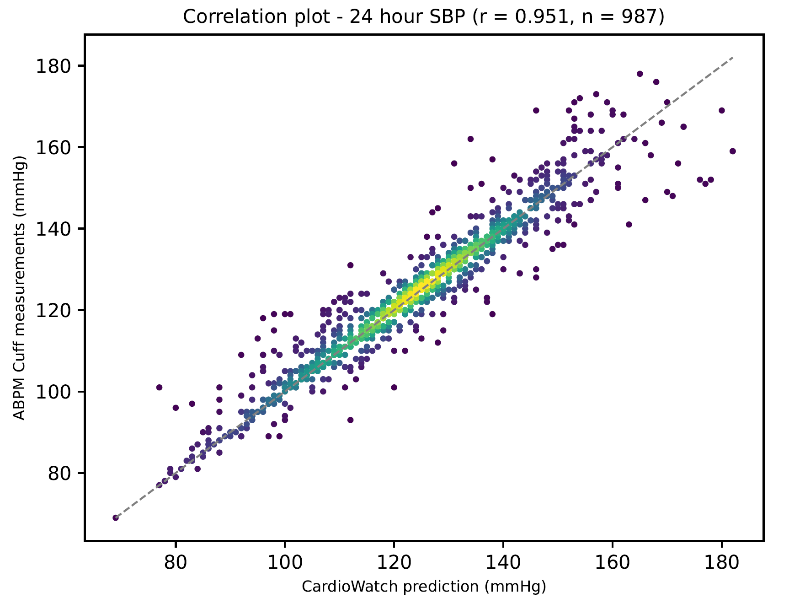

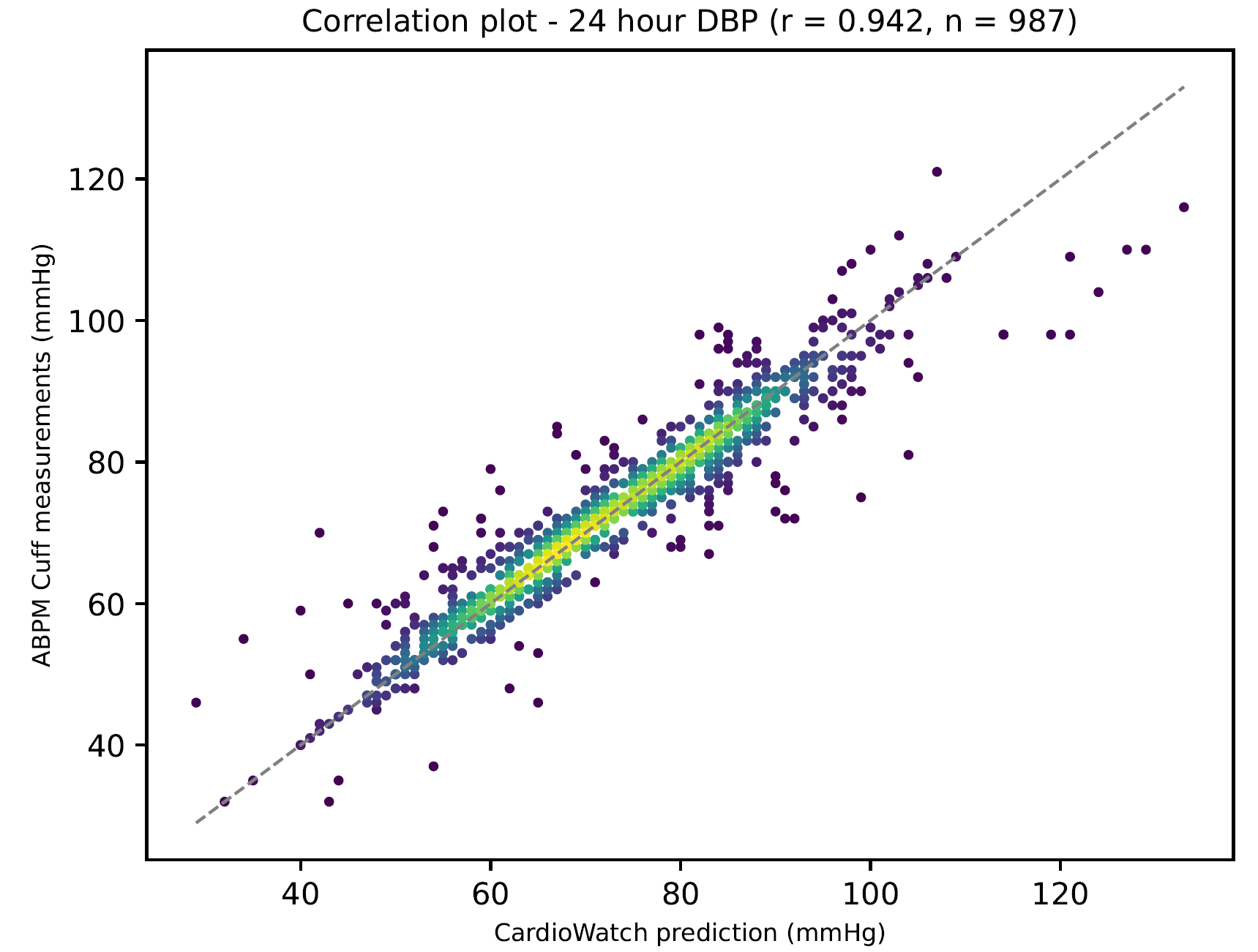

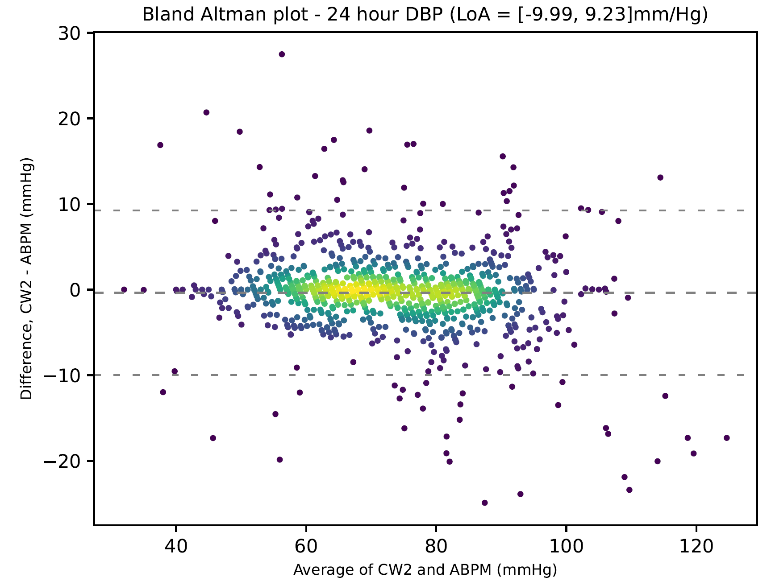

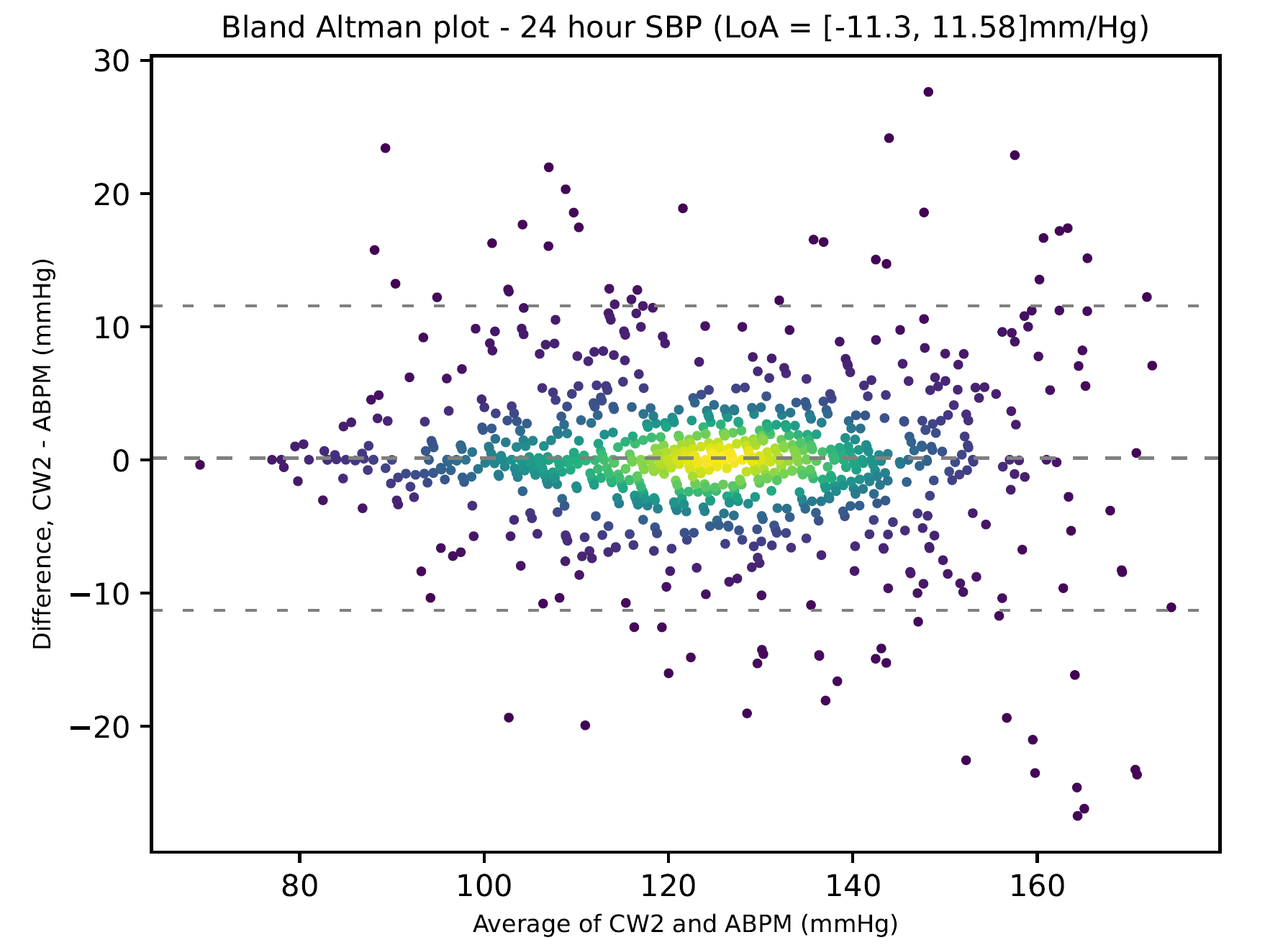


**Supplementary figure 2.** Visualatisation of Bland-Altman analaysis for the investigational and reference **a)** systolic and **b)** diastolic blood pressure as well as the correlation between the investigational and reference **c)** systolic and **d)** diastolic blood pressure, both across the entire 24-hour measurement period using density plots (high to low density = yellow – green – blue – purple).

**B**

**C**

**D**

**A**


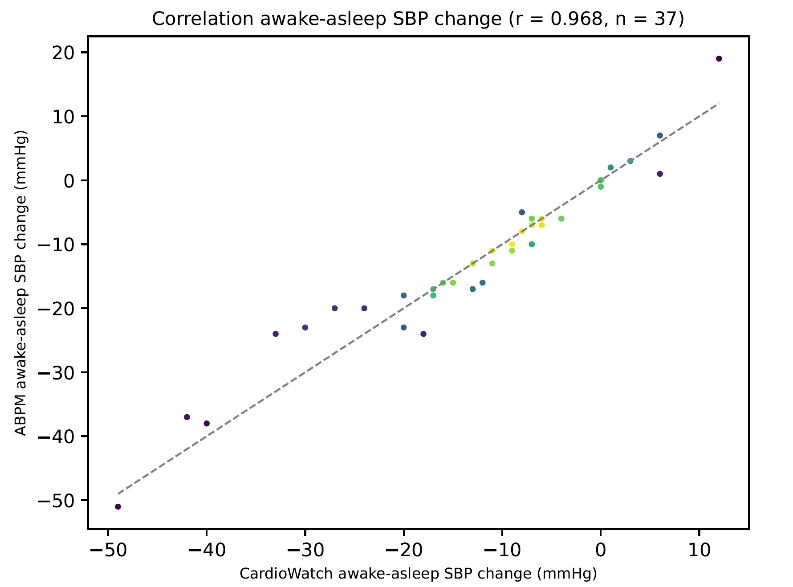

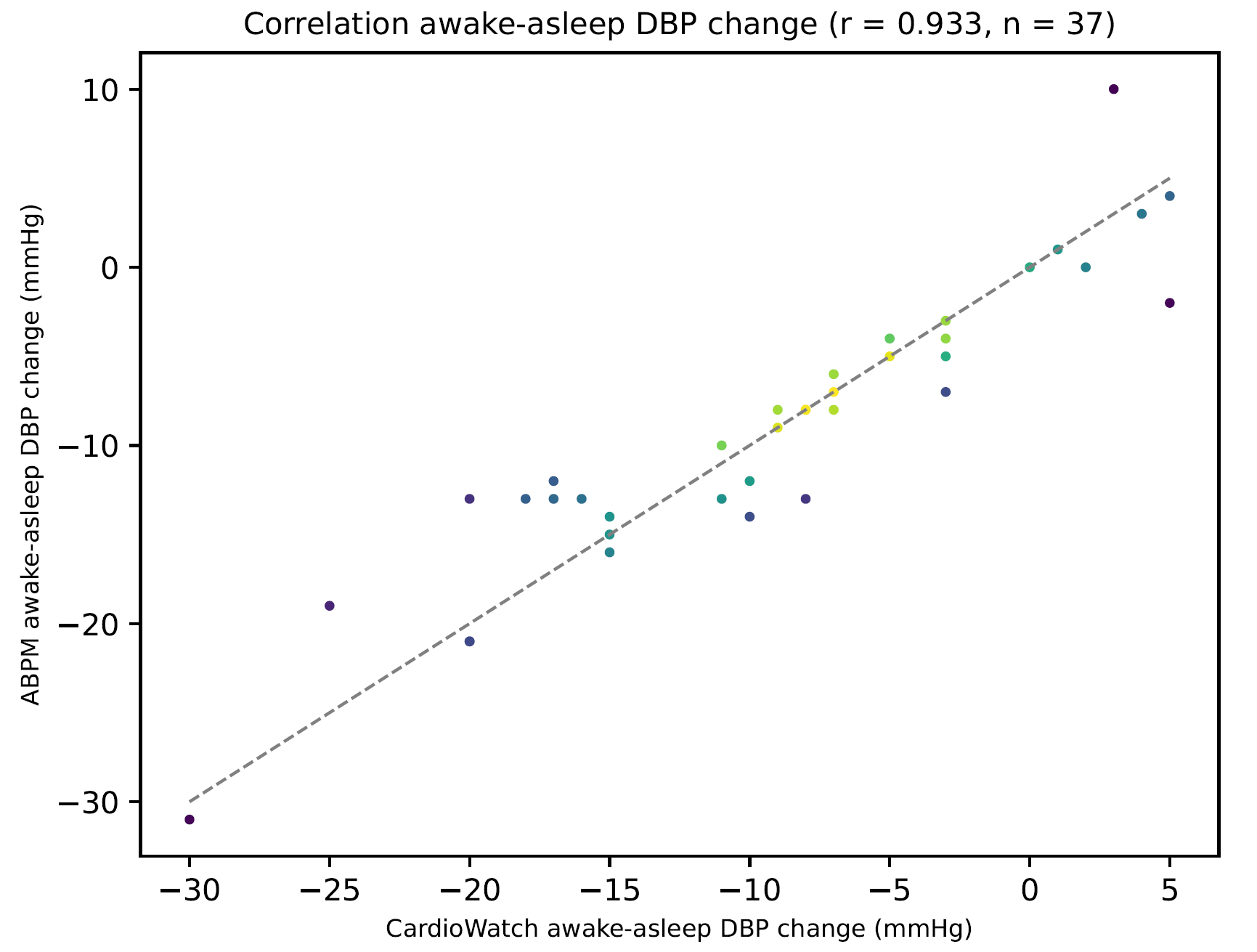

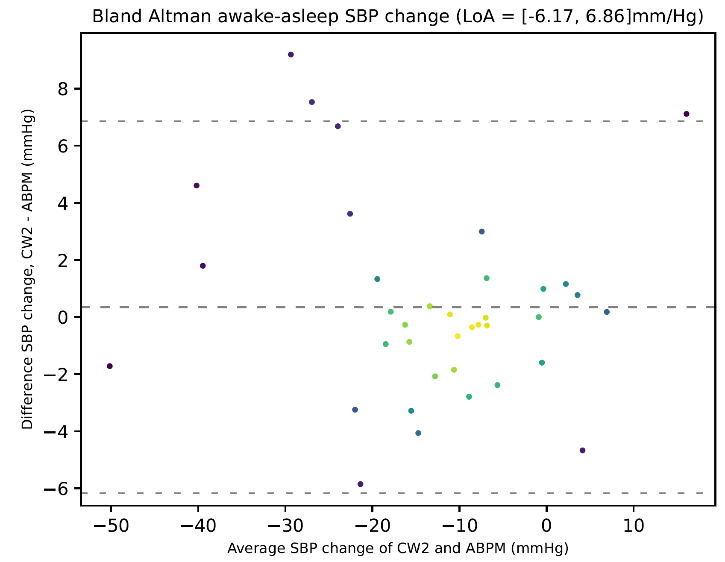

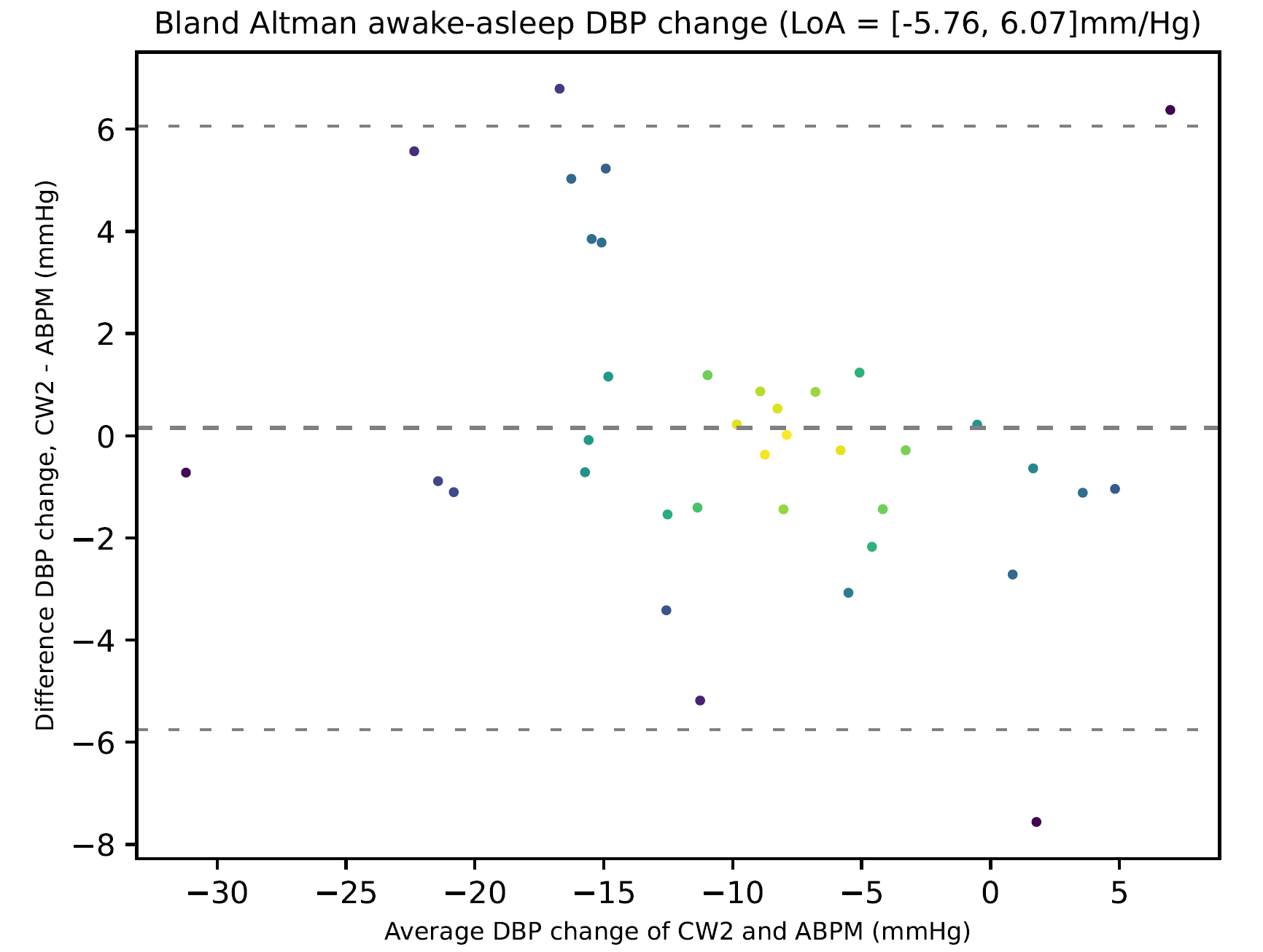


**A**

**B**

**C**

**D**

**Supplementary figure 3.** Visualisation of Bland-Altman analysis for the investigational and reference awake-asleep **a)** systolic and **b)** diastolic blood pressure difference as well as the correlation between the investigational and reference awake-asleep **c)** systolic and **d)** diastolic blood pressure difference (awake-asleep test) using density plots (high to low density = yellow – green – blue – purple).
